# A Machine Learning Approach to Study Demographic Alterations in Honeybee Colonies Using SDS–PAGE Fingerprinting

**DOI:** 10.3390/ani11061823

**Published:** 2021-06-18

**Authors:** Riccardo Cabbri, Enea Ferlizza, Elisa Bellei, Giulia Andreani, Roberta Galuppi, Gloria Isani

**Affiliations:** 1 Department of Veterinary Medical Sciences, University of Bologna, Via Tolara di Sopra 50, 40064 Ozzano Emilia, Italy; rccabbri@gmail.com (R.C.); giulia.andreani2@unibo.it (G.A.); roberta.galuppi@unibo.it (R.G.); gloria.isani@unibo.it (G.I.); 2 Department of Experimental, Diagnostic and Specialty Medicine, University of Bologna, Via Belmeloro 8, 40126 Bologna, Italy; 3 Department of Surgery, Medicine, Dentistry and Morphological Sciences with Transplant Surgery, Oncology and Regenerative Medicine Relevance, University of Modena and Reggio Emilia, via del Pozzo 71, 41124 Modena, Italy; elisa.bellei@unimore.it

**Keywords:** *Apis mellifera*, nurses, foragers, colony depopulation, haemolymph proteins

## Abstract

**Simple Summary:**

Honeybees are vital pollinators for the human food chain. Colony depopulation is a serious threat to Apis mellifera populations and unfortunately it is also one of the most elusive and difficult to study. This research deals with the problem at its foundation: population imbalances. The proposed method allows to discriminate, with remarkably good performances, precocious foragers from proper aged ones using SDS-PAGE patterns of haemolymph proteins. Implications and future perspectives are discussed.

**Abstract:**

Honeybees, as social insects, live in highly organised colonies where tasks reflect the age of individuals. As is widely known, in this context, emergent properties arise from interactions between them. The accelerated maturation of nurses into foragers, stimulated by many negative factors, may disrupt this complex equilibrium. This complexity needs a paradigm shift: from the study of a single stressor to the study of the effects exerted by multiple stressors on colony homeostasis. The aim of this research is, therefore, to study colony population disturbances by discriminating overaged nurses from proper aged nurses and precocious foragers from proper aged foragers using SDS-PAGE patterns of haemolymph proteins and a machine-learning algorithm. The KNN (K Nearest Neighbours) model fitted on the forager dataset showed remarkably good performances (accuracy 0.93, sensitivity 0.88, specificity 1.00) in discriminating precocious foragers from proper aged ones. The main strength of this innovative approach lies in the possibility of it being deployed as a preventive tool. Depopulation is an elusive syndrome in bee pathology and early detection with the method described could shed more light on the phenomenon. In addition, it enables countermeasures to revert this vicious circle.

## 1. Introduction

The effect of the recently reported decline in insects [1] and particularly wild pollinators [2] is alarming due to the dependence of agriculture and wild plants on pollination services. Moreover, the same drivers of the abovementioned insects decline also affect managed *Apis mellifera* colonies [3]. Given the multifactorial nature of the phenomenon [4], a traditional reductionist approach could face difficulties in tackling and preventing the issue. Thus, an innovative and holistic perspective to objectively evaluate the health status could overcome these limits and give operators some perspective about the current situation and prognosis of the colonies.

Honeybees are social insects, living in colonies often referred to as superorganisms in order to highlight the high level of organisation achieved. A superorganism is a collection of individuals that are mutually dependent and together possess the functional organisation of an organism. The strengths of this level of organisation are the emergent properties arising from the interactions between individual members of the colony, such as thermoregulation, comb construction or foraging behaviour [5].

The adult members of the colony are mainly represented by female workers, characterised by three different phenotypes: nurse bees, foragers and diutinus workers [6]. While the latter are reared in peculiar conditions, naturally occurring in autumn (in temperate climates) or artificially obtained during the active season through brood interruption [6,7,8,9], the first two phenotypes are age-related (so called “age polyethism”). Workers act as nurse bees within 3–4 days of emergence [10]. They have hypertrophic hypopharyngeal glands and fat bodies for royal jelly production and brood caring. In addition, nurse bees have a high number of circulating haemocytes [11].

Nurse bees eventually become foragers [12], responsible for the nutrient supply to the colony. Contrary to nurse bees, foragers are characterised by atrophy of hypopharyngeal glands and fat body [11] and by a lower cellular immunity due to haemocyte apoptosis [13]. The transition from nurse to forager is not direct, since other tasks are performed by the bees after feeding larvae but prior to explore the outside of the hive. In this paper, we refer to “hive bees” to indicate bees performing tasks inside the hive.

The transition from hive bees to foragers is socially regulated trough a feedback loop between vitellogenin and juvenile hormone, key proteins in foraging regulation [14,15]. The timing of this transition is not fixed and can be anticipated or postponed following the colony needs [16,17]. The accelerated maturation of hive bees into precocious foragers is favoured by many stimuli: undernutrition, wax deprivation, lack of pollen and forager loss [18,19,20,21]. However, some detrimental effects can occur, such as lower flight performances that have been reported in precocious foragers compared to normal aged foragers [22]. Interestingly, the action of pathogens can also trigger this behaviour: both *Nosema* spp. and *Varroa destructor* have been proven to cause precocious foraging [23,24]. Moreover, a recent research [25] identified a common host response to different pathogens (including *Nosema* spp. and *Varroa*, Israeli Acute Paralysis Virus, Black Queen Cell Virus and Deformed Wing Virus), including a decrease in the expression of vitellogenin.

This evidence may suggest the presence of a common pathway of colony depopulation, as recently proposed by Perry et al. [26], and therefore, the usefulness of a paradigm shift: from the study of a single stressor to the study of the effects exerted by multiple stressors to the colony homeostasis. To date, only Aluax et al. [27] proposed a method to study demographic alterations in the colony, based on the estimation of biological age trough gene expression of vitellogenin and adipokinetic hormone receptor. However, a more comprehensive approach is needed. Proteomics enables separation and identification of a selected set of proteins, e.g., haemolymph proteins, and can provide important information about its complexity and variations.

The aim of this research is to study colony population unbalance by discriminating overaged nurses from proper aged nurses and precocious foragers from proper aged foragers through the study of the electrophoretic pattern of haemolymph proteins and a machine-learning algorithm. Machine learning is a supervised learning approach where an algorithm is trained on a dataset consisting of predictors and dependent variables in order to formulate prediction rules. These rules are then exploited to predict the dependent variables knowing only the predictors. In this case, the predictors are represented by the intensities of the protein bands, found at specific migration distances on the gel; the dependent variable is a categorical binary outcome: precocious forager and proper aged forager or overaged nurse and proper aged nurse.

## 2. Materials and Methods

### 2.1. Single Cohort Colonies Setup and Sample Collection

This research used “single cohort colonies” (SCCs). These colonies consist of a variable number of same aged workers, obtained by controlled eclosion in an incubator, and a fertile queen. In these colonies, 8 to 10 days after eclosion, some workers start foraging (precocious foragers) and some others initiate brood caring (proper aged nurses); 21 days after eclosion, foragers of proper age start foraging while brood caring relies on overaged nurses [28]. 

Two trials with SCCs were conducted in June–July and in September–October. The reason behind the different timing in the two replicates is to collect data from different physiological moments of the colonies, and thus, to better generalise the results. Both experiments took place in a dedicated apiary at the Department of Veterinary Medical Sciences of the University of Bologna, Italy.

To obtain workers of the same age to populate the SCCs, the following protocol was employed:

- On day −21, four mated sister queens were caged on four different combs, drawn from organic-certified residue free wax, and placed inside four fully developed and healthy colonies;
- On day −19 the queens were removed from the cages, in order to have a maximum difference of 48 h among the brood laid;
- On day +1, newly eclosed workers were gently brushed from the combs, mixed to eliminate the mother colony factor and used to prepare the two SCCs. Each SCCs was made with 250 g of *Apis mellifera ligustica* bees (equivalent to approximately 2500 individuals), one queen (of the same subspecies) and two combs drawn from the same wax mentioned above: one empty and one with plenty of honey and pollen. The SCCs were kept closed in a protected and shaded environment to allow complete maturation of the workers;
- On day +3, 3 days post-eclosion, the SCCs were moved to the outdoor apiary.

With this setup, four categories of bees were obtained: precocious foragers (*n* = 28) and proper aged nurses (*n* = 35) sampled on days +8 to +10, and overaged nurses (*n* = 36) and proper aged foragers (*n* = 35) sampled on day +21. The queens were added on day +1 through an introduction cage, sealed on one end with candy. It took a couple days to the workers to free her so that, on day +21, no newly eclosed bees were present in the hive potentially hindering the sampling process. In order to increase the specificity of sampling, only bees displaying the behaviour of feeding larvae were sampled as nurses and only bees leaving the hive (caught with a home-made apparatus hanging in front of the flight entrance) outside the central hours of the day, when orientation flights usually take place, were sampled as foragers.

### 2.2. Haemolymph Collection and SDS-PAGE Electrophoresis

Two microliters of haemolymph were drawn from each bee with a graduated glass microcapillary according to Cabbri et al. (2018) [8] and stored at −80 °C. For each of the 134 samples analysed, 3 μg of proteins were loaded and separated with 4–12% gradient gels, in MOPS buffer (NuPAGE, Thermo Fisher Scientific, Waltham, MA, USA). The gels were stained with Coomassie G250 compatible with mass spectrometry analysis, digitalised by ChemiDoc™MP (BioRad, Hercules, CA, USA) and the pherograms were obtained using the ImageLab 5.2.1 software (BioRad, Hercules, CA, USA). Protein identification by mass spectrometry was carried out according to Cabbri et al. (2018) [7].

### 2.3. Data Preparation

Gel images were imported in Fiji, a software based on ImageJ 1.52i [29], coupled with the Bioformats 6.0.0 plugins in order to read the proprietary .scn files. The lanes of each gel were manually delimited drawing a segmented line through the centre (from the loading well to the end of the gel), adding it as a ROI (Region of Interest) and specifying the width. Afterwards, the electropherograms were plotted using the Multi-plot command and exported to an Excel spreadsheet. In this file, columns represent the samples, rows represent the distances from the loading well. The intersection of the two contains the intensities of the pixels in that area of the gel. The data of each gel were collected in a comprehensive database and analysed with the statistical software R 3.6.0 [30] and the RStudio IDE [31].

The *distance* variable was binned in order to reduce the complexity, and thus, the computation time of the dataset. One hundred intervals were created, and the corresponding intensities were averaged. The *distance* variable was created using the median value of every interval. After this processing, the resolution was about 650 µm.

To compensate for the differences in migration patterns of the various gels, the GCalignR [32] library was used. The areas of the pherograms below 5000 µm and exceeding 45,000 µm of migration distance were excluded from the alignment in order to avoid high noise zones. The aligned data matrix was used for the subsequent analysis.

### 2.4. Statistical Analysis

Two different datasets were prepared, one containing the nurses’ data and the other containing the foragers’ data. Each dataset was randomly split in a training set (75% of the cases) and a test set (25% of the cases).

Recursive feature elimination (RFE) with random forests function was used to select relevant variables in the foragers’ and nurses’ datasets, separately (selection made with rknn 1.2-1 package). A conservative approach based on the empirical “one in ten rule” was used to choose the maximum number of features to retain. Considering a number of cases in the train set of approximately 50, the maximum number was set to 5.

To avoid selection bias, the external validation was achieved through 10-fold cross-validation [33]. Considering the deviation of data from normality (Shapiro–Wilk normality test), non-parametric algorithms were chosen. Three different models based on three different algorithms were fit using the train dataset: Support Vector Machines with Linear Kernel (SVM), k-Nearest Neighbours (KNN) and Random Forest (RF). Cross-validation and model fitting was achieved with caret 6.0-78 package [34].

The performances of the models were evaluated by repeating 10 times a 10-fold cross-validation considering as parameters the AUC (Area Under the Curve), Sensitivity and Specificity; differences were computed, then a *t*-test was used to evaluate the null hypothesis that there is no difference between models.

The generalisation error was then assessed on the test set by building a confusion matrix and computing again the AUC, Accuracy (with 95% CI), Sensitivity and Specificity for each model. The null Accuracy was compared with the obtained Accuracy and a *p*-value was computed to know if the classifier is significantly better than a random classifier. A *p*-value < 0.05 was considered as significant.

## 3. Results

### 3.1. Nurses

**Figure 1:**
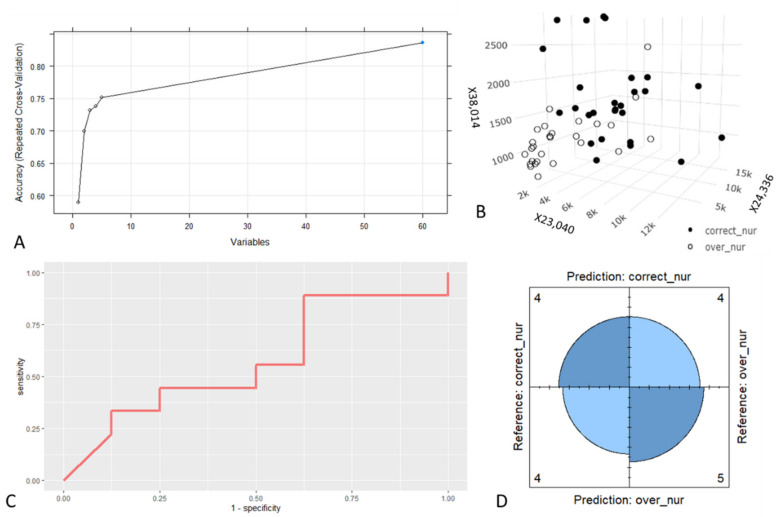
Nurses’ data. (**A**) Relation between the mean Accuracy of the model obtained with the nurse training dataset (assessed through Repeated Cross-Validation) and the number of variables used. (**B**) Three-dimensional plot showing the relation between the intensity values of the three most informative variables and the nurse category in the training dataset. (**C**) ROC curve computed with the prediction of the RF model on the nurse test dataset. (**D**) Four-fold plot showing the results of the confusion matrix produced with the prediction of the RF model on the nurse test dataset.

The results obtained on the nurses’ dataset showed a negligible improvement in accuracy with a number of variables exceeding three (Figure 1A; Table S1) in the 1:5 range chosen to limit overfitting. For this reason, the first three variables were chosen to build the models. Those with the highest ranking were: X23,040, X24,336 and X38,014 (Figure 1B). Performances obtained through resampling the training dataset are summarised in Table 1. The differences in mean AUCs were not statistically significant, while the mean sensitivity achieved through the KNN model was significantly higher (*p* < 0.05) than the sensitivity of the SVM model. Regarding mean specificity, the value of the KNN model is significantly lower (*p* < 0.05) than that of the other models. The best overall performance is obtained with the RF model. Performances calculated on the test set for the RF model are summarised in Table 2 and in the ROC curve (Figure 1C). However, the model on the test set was not able to discriminate the two categories, as shown in the four-fold plot (Figure 1D).

### 3.2. Foragers

**Figure 2:**
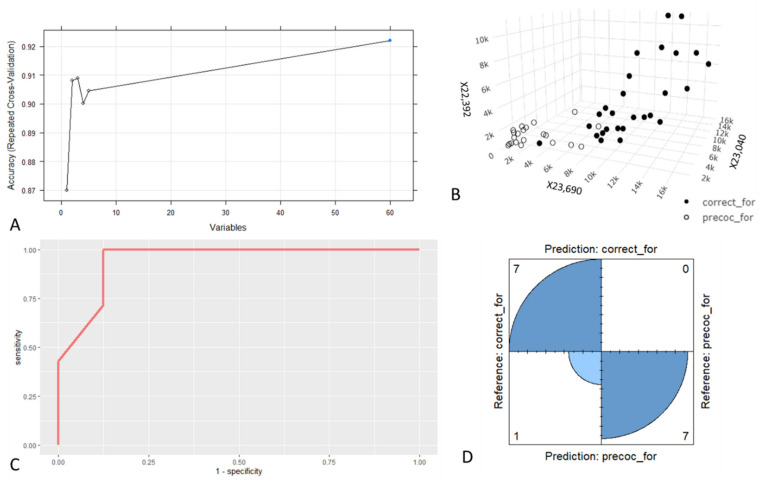
Foragers’ data. (**A**) Relation between the mean Accuracy of the model obtained with the forager training dataset (assessed through Repeated Cross-Validation) and the number of variables used. (**B**) Three-dimensional plot showing the relation between the intensity values of the three most informative variables and the category of the foragers in the train dataset. (**C**) ROC curve computed with the prediction of the RF model on the forager test dataset. (**D**) Four-fold plot showing the results of the confusion matrix produced with the prediction of the RF model on the forager test dataset.

The results obtained on the foragers’ dataset showed a negligible improvement in accuracy with a number of variables exceeding a value of 3 (Figure 2A), so the first three variables were chosen to build the models. Those with the highest ranking were: X22,392, X23,040 and X23,690 (Figure 2B). Performances obtained through resampling on the training dataset are summarised in Table 1. The differences in mean AUCs were not statistically significant, while the mean sensitivity achieved through the RF model was significantly lower (*p* < 0.05) than the sensitivity obtained by the other models. Regarding the mean specificity, no significant differences were found between models. The best overall performances were obtained with SVM and KNN, the latter was preferred as it was less computationally intensive.

Performances calculated on the test set for the KNN model are summarised in Table 2 and in the ROC curve (Figure 2C). The model on the test dataset was able to discriminate the two categories with 100% prediction accuracy in discriminating precocious foragers (Figure 2D).

**Figure 3:**
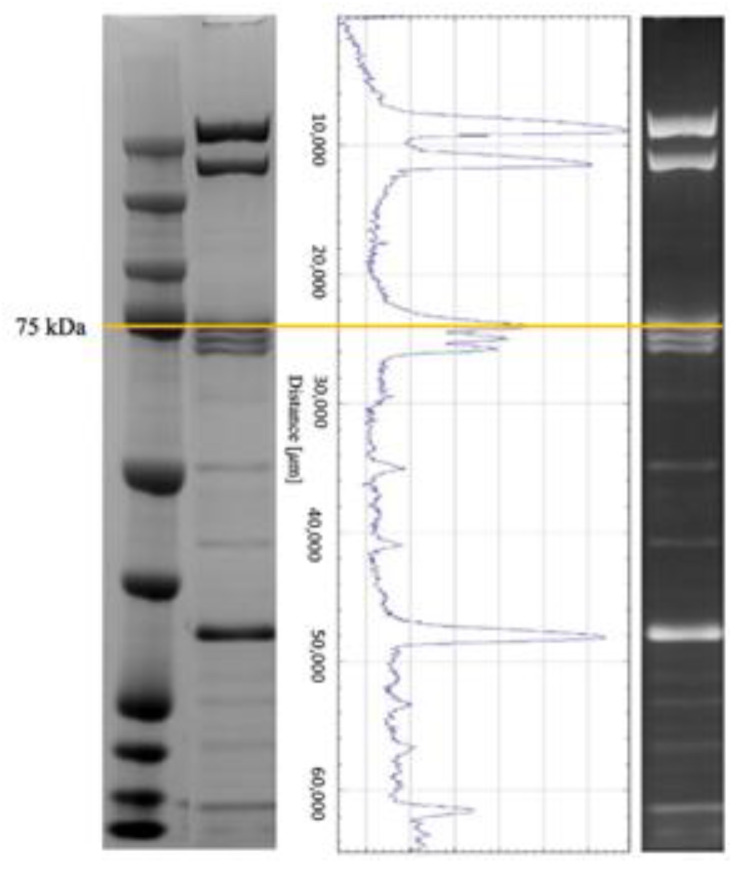
From left to right: molecular weight marker; representative lane of a gel; pherogram of the same lane as acquired through Fiji software; lane with inverted colours as needed for image analysis. The line highlights the zone of the gel, comprising a range of 22,064–24,015 µm, including the band cut for mass identification.

The features chosen for the foragers model correspond to three contiguous zones of the gel, comprising 22,064–24,015 µm of the migration distance, containing a well-defined protein band with an apparent molecular mass of 75 kDa (Figure 3). This band was cut from the gel and proteins were identified using mass spectrometry. The search in the UniProt database resulted in the identification of three proteins (Table 3): the highest score and number of matches were obtained for apolipophorin, followed by leucine-rich repeat-containing protein 15 (LRRC15) and transferrin.

## 4. Discussion

The timing of nurse to forager maturation is of capital importance in determining the longevity of honeybees [28]. A wide variety of stress factors exert an influence on this timing, and thus, greatly impact the colony population dynamics, possibly leading to colony depopulation [26]. For this reason, a machine learning method was developed with the aim of discriminating bees whose age matches the role held in the colony from bees whose age does not. In this study, a proteomic fingerprinting approach to gel analysis was chosen to explore the overall contribution of the main haemolymph proteins, without the bias related to an a priori selection.

Overaged nurses are not functionally equivalent to the proper aged ones; in fact, the degeneration of mandibular glands, which occurs in old nurses, leads to phenotypic differences in the reared workers, which exhibit higher ovary development [35]. Higher ovary development in workers was linked to a suboptimal foraging behaviour, determining an overall decrease in the performance of the colony [36]. Therefore, it is possible that workers reared by overaged nurses become less competent foragers. The RF algorithm indicated the variables X23,040, X24,336 and X38,014 as the most relevant to discriminate overaged nurses from proper aged ones. The maximum accuracy was achieved with all 60 variables, but in the range 1:5, which was chosen to avoid overfitting the model, no significant improvement of accuracy was achieved using a number of predictors exceeding three. Despite the good performances exhibited in the train dataset, a conspicuous generalisation error affected the chosen RF model as shown by the poor predictive performance on the test dataset. The accuracy obtained is not significantly different from the null accuracy and this model has no predictive power nor practical use.

Better results were obtained with the foragers’ dataset. The RF algorithm indicated the variables X23,690, X23,040 and X22,392 as the most relevant to discriminate the two categories. The excellent performances of the KNN model in the training dataset were confirmed in the test dataset. As seen in the four-fold plot, the model was able to discriminate with 100% accuracy (7/7) the precocious foragers in the test set.

The features chosen for the foragers model correspond to three contiguous zones of the gel, comprising 22,064–24,015 µm of the migration distance, containing a band of an apparent molecular mass of 75 kDa. The proteins identified in this band using mass spectrometry were apolipophorin, leucine-rich repeat-containing protein 15 (LRRC15) and transferrin.

Apolipophorins are the major lipoproteins in insects and their presence in haemolymph is closely related to lipid mobilisation [37]. An apolipophorin showing this unusually low molecular mass has been described as apolipophorin II (ApoLp-II) in *Manduca sexta* [38]. Apolipophorin I (ApoLp-I) and Apolipophorin II (ApoLp-II) are produced from a post-translational cleavage of a precursor protein, apolipophorin II/I (ApoLp-II/I) [39]. To date, no studies deal with the specific function of ApoLp-II in *A. mellifera*. However, Wen et al. [40] recently demonstrated an upregulation of Ap-apoLp-II/I gene expression in response to bacterial challenge and a novel role for Ap-apoLp-II/I in regulating the prophenoloxidase activation system in *Antheraea pernyi*. The identification of a putative ApoLp-II in haemolymph of forager honeybees is intriguing and its involvement in the immune response can also be hypothesised in *A. mellifera*.

The LRRC15 protein belongs to a ubiquitous protein superfamily characterised by the presence of leucine-rich repeats (LRR). LRR proteins are involved in a wide variety of biological processes, from signal transduction to disease resistance and immune response [41]. In particular, in *Drosophila melanogaster*, LRR proteins contribute to the response to insecticides and regulate the immune response and the NF-kB signalling pathway. Additionally, in honeybees, it has been reported that exposure to sub-lethal doses of the neonicotinoid clothianidin enhances the transcription of the gene encoding an LRR protein, reduces immune defences and promotes the proliferation of the deformed wing virus [42].

Transferrins (Tsfs) are monomeric iron-binding proteins ubiquitous in metazoans. There are three transferrin homologs in *Drosophila*: Tsf1, Tsf2 and Tsf3. Tsf1 is considered the insect counterpart of mammalian serum transferrin. This protein is synthesised in the fat body, secreted into the haemolymph and involved in trafficking iron from the gut to the fat body where the metal is stored bound to ferritin [43]. The role of Tsf1 seems not limited to iron homeostasis. Recently, Iatsenko et al. [44] reported that infections determined a hypoferremic response in *D. melanogaster* due to iron withdrawal from the haemolymph and storage in the fat body, suggesting that Tsf1 mediates the nutritional immunity in the fly.

Overall, in addition to their specific biological tasks, ApoLp-II, LRRC15 protein and transferrin identified in the haemolymph of foragers could be involved in the activity of the immune system, suggesting a possible involvement of the immune response in the complex and still little explored mechanisms related to precocious foraging, bee health decline and subsequent colony unbalance. In eusocial insects, the immune system plays an important role in ensuring colony survival and the transition between nurses and foragers is a crucial step to maintain colony homeostasis. A recent study demonstrated that honeybee foragers exhibit greater expression of genes associated with the immune response than do nurse bees, suggesting that these genes are involved in the first line of defence against pathogens [45]. In addition to minor foraging experience, less immunocompetent precocious foragers could transfer pathogens within the hive leading to an increased risk of colony decline or mortality.

## 5. Conclusions

This work deals with the often-neglected problem of demographic alterations in honeybee colonies. The KNN model fitted on the foragers’ dataset showed a remarkably good predictive accuracy, making it an interesting candidate to monitor population imbalances in the colony. Given the experimental setup, a trial on fully developed colonies artificially deprived of foragers is needed to further validate this tool. Moreover, the study of a protocol using a pool of bees instead of single insects would be useful to reach an affordable solution for beekeepers and veterinarians operating in this field.

The main strength of this approach lies in the possibility of it being deployed as a preventive tool. Depopulation is an elusive syndrome in bee pathology because it leaves behind no bees to sample. Early detection with the method described herein could enable countermeasures to revert the vicious circle.

At the moment, the main weaknesses are the need of trained personnel for haemolymph collection and the high processing time required. Research on haemolymph collection methods could unravel easier and more convenient techniques.

## Figures and Tables

**Table 1:** Performances of the models assessed through resampling on the training dataset.

	Nurses			Foragers		
Model	AUC	Sensitivity	Specificity	AUC	Sensitivity	Specificity
SVM	0.87 _(a)_	0.76 _(a)_	0.83 _(a)_	0.98 _(a)_	0.96 _(a)_	0.86 _(a)_
KNN	0.83 _(a)_	0.9 _(b)_	0.67 _(b)_	0.98 _(a)_	0.96 _(a)_	0.87 _(a)_
RF	0.82 _(a)_	0.82 _(ab)_	0.75 _(ab)_	0.96 _(a)_	0.89 _(b)_	0.90 _(a)_

Different lowercase letters between rows within a column indicate statistically significant (*p* < 0.05) difference. Support Vector Machines with Linear Kernel (SVM), k-Nearest Neighbours (KNN) and Random Forest (RF); area under curve (AUC).

**Table 2:** Performances of the selected model on the test set.

	Nurses	Foragers
	RF	KNN
Accuracy	0.53	0.93
Accuracy Lower	0.28	0.68
Accuracy Upper	0.77	1
Accuracy Null	0.53	0.53
Accuracy *p*-Value	0.6	0.00113
Sensitivity	0.5	0.88
Specificity	0.56	1
AUC	0.57	0.95

**Table 3:** Identification table.

Accession ^1^	Description	Mass (kD) ^2^	Score ^3^	Pep ^4^	Pep (sig) ^5^	Seq ^6^	Seq (sig) ^7^	Protein Homologous ^8^	% Identity ^9^	Species ^10^
A0A088AS56	Uncharacterised protein	369	3848	410	224	48	35	Apolipophorins	91.8	*Apis cerana*
A0A088AQB0	Uncharacterised protein	76	1001	98	59	19	13	Leucine-rich repeat-containing protein 15	98.4	*Apis cerana*
A0A088AFH7	Transferrin	80	340	55	24	19	9		100	*Apis mellifera*

^1^ Protein entry name from the UniProt knowledge database. ^2^ Theoretical protein molecular mass. ^3^ The highest scores obtained with the Mascot search engine. ^4^ Peptides: total number of peptides matching the identified proteins. ^5^ Significant peptides: total number of significant peptides matching the identified proteins. ^6^ Sequences: total number of distinct sequences matching the identified proteins. ^7^ Significant sequences: total number of significant distinct sequences matching the identified proteins. ^8^ Protein homologous after BLAST searching in the Uniprot knowledge database. ^9^ Percentage of identical sequences between the identified and the homologous protein as reported after BLAST searching in the Uniprot knowledge database. ^10^ Species of the homologous protein.

## Data Availability

The data presented in this study are openly available in FigShare at https://doi.org/10.6084/m9.figshare.14792124.v1, https://doi.org/10.6084/m9.figshare.14792118.v1, https://doi.org/10.6084/m9.figshare.14792073.v1, https://doi.org/10.6084/m9.figshare.14792049.v1, https://doi.org/10.6084/m9.figshare.14792046.v1, https://doi.org/10.6084/m9.figshare.14792037.v1 (accessed date for all links is 18 June 2021).

## Supplementary Materials

The following are available online at https://www.mdpi.com/article/10.3390/ani11061823/s1. Table S1: Mean accuracy and standard deviation (SD) of the model obtained with the train dataset (assessed through Repeated Cross-Validation) and number of variables used.
