# Supplementary material for: A Machine Learning Approach to Study Demographic Alterations in Honeybee Colonies Using SDS–PAGE Fingerprinting
Source: Animals (Basel). 2021 Jun 18;11(6):1823. doi: 10.3390/ani11061823 (PMC8233723; doi:10.3390/ani11061823)
Supplement: Supplementary file 1 [file animals-11-01823-s001.zip › Cabbri_et_al_honeybee_demography_supplementary.pdf]

Table S1. Mean accuracy and standard deviation (SD) of the model obtained with the train dataset (assessed through Repeated Cross-Validation) and number of variables.

NURSES			FORAGERS		
Variables	Accuracy	SD	Variables	Accuracy	SD
1	0.59	0.18	1	0.87	0.13
2	0.7	0.17	2	0.91	0.14
3	0.73	0.16	3	0.91	0.13
4	0.74	0.16	4	0.9	0.14
5	0.75	0.17	5	0.9	0.13
60	0.84	0.16	60	0.92	0.11
